# Supplementary material for: Unveiling the Bioactive Potential of the Invasive Jellyfish Phyllorhiza punctata Through Integrative Transcriptomic and Proteomic Analyses
Source: Biomolecules. 2025 Aug 4;15(8):1121. doi: 10.3390/biom15081121 (PMC12383608; doi:10.3390/biom15081121)
Supplement: Supplementary file 1 [file biomolecules-15-01121-s001.zip › Suplementary Material/FileS3/PEPFOLD4/XP_065051325_11252/clusters.html]

 PEP-FOLD clusters


Download archive of all models  here   

|  |  |  |
| --- | --- | --- |
| # | file | sOPEP |
| # cluster 1 - Size: 19 |  |  |
|  | XP\_065051325\_11252-model1 | -11.5681 |
|  | XP\_065051325\_11252-model1.1 | -11.5681 |
|  | XP\_065051325\_11252-model1.2 | -11.5681 |
|  | XP\_065051325\_11252-model1.3 | -11.4957 |
|  | XP\_065051325\_11252-model1.4 | -11.4957 |
|  | XP\_065051325\_11252-model1.5 | -11.4957 |
|  | XP\_065051325\_11252-model1.6 | -11.4957 |
|  | XP\_065051325\_11252-model1.7 | -11.4061 |
|  | XP\_065051325\_11252-model1.8 | -11.4061 |
|  | XP\_065051325\_11252-model1.9 | -11.3337 |
|  | XP\_065051325\_11252-model1.10 | -11.3337 |
|  | XP\_065051325\_11252-model1.11 | -11.3337 |
|  | XP\_065051325\_11252-model1.12 | -11.3269 |
|  | XP\_065051325\_11252-model1.13 | -11.3269 |
|  | XP\_065051325\_11252-model1.14 | -11.3269 |
|  | XP\_065051325\_11252-model1.15 | -11.3269 |
|  | XP\_065051325\_11252-model1.16 | -11.2952 |
|  | XP\_065051325\_11252-model1.17 | -11.2025 |
|  | XP\_065051325\_11252-model1.18 | -11.1657 |
| # cluster 2 - Size: 36 |  |  |
|  | XP\_065051325\_11252-model2 | -11.5654 |
|  | XP\_065051325\_11252-model2.1 | -11.5654 |
|  | XP\_065051325\_11252-model2.2 | -11.5654 |
|  | XP\_065051325\_11252-model2.3 | -11.5654 |
|  | XP\_065051325\_11252-model2.4 | -11.5654 |
|  | XP\_065051325\_11252-model2.5 | -11.5654 |
|  | XP\_065051325\_11252-model2.6 | -11.5654 |
|  | XP\_065051325\_11252-model2.7 | -11.3851 |
|  | XP\_065051325\_11252-model2.8 | -11.3851 |
|  | XP\_065051325\_11252-model2.9 | -11.3805 |
|  | XP\_065051325\_11252-model2.10 | -11.3655 |
|  | XP\_065051325\_11252-model2.11 | -11.3655 |
|  | XP\_065051325\_11252-model2.12 | -11.3655 |
|  | XP\_065051325\_11252-model2.13 | -11.3655 |
|  | XP\_065051325\_11252-model2.14 | -11.3655 |
|  | XP\_065051325\_11252-model2.15 | -11.3655 |
|  | XP\_065051325\_11252-model2.16 | -11.3567 |
|  | XP\_065051325\_11252-model2.17 | -11.3567 |
|  | XP\_065051325\_11252-model2.18 | -11.3567 |
|  | XP\_065051325\_11252-model2.19 | -11.3567 |
|  | XP\_065051325\_11252-model2.20 | -11.3567 |
|  | XP\_065051325\_11252-model2.21 | -11.3567 |
|  | XP\_065051325\_11252-model2.22 | -11.3567 |
|  | XP\_065051325\_11252-model2.23 | -11.3567 |
|  | XP\_065051325\_11252-model2.24 | -11.3567 |
|  | XP\_065051325\_11252-model2.25 | -11.3567 |
|  | XP\_065051325\_11252-model2.26 | -11.3567 |
|  | XP\_065051325\_11252-model2.27 | -11.2889 |
|  | XP\_065051325\_11252-model2.28 | -11.2889 |
|  | XP\_065051325\_11252-model2.29 | -11.2773 |
|  | XP\_065051325\_11252-model2.30 | -11.2693 |
|  | XP\_065051325\_11252-model2.31 | -11.2305 |
|  | XP\_065051325\_11252-model2.32 | -11.1653 |
|  | XP\_065051325\_11252-model2.33 | -11.1342 |
|  | XP\_065051325\_11252-model2.34 | -11.1342 |
|  | XP\_065051325\_11252-model2.35 | -11.0581 |
| # cluster 3 - Size: 1 |  |  |
|  | XP\_065051325\_11252-model3 | -11.3591 |
| # cluster 4 - Size: 4 |  |  |
|  | XP\_065051325\_11252-model4 | -11.3472 |
|  | XP\_065051325\_11252-model4.1 | -11.3197 |
|  | XP\_065051325\_11252-model4.2 | -11.3197 |
|  | XP\_065051325\_11252-model4.3 | -11.3197 |
| # cluster 5 - Size: 3 |  |  |
|  | XP\_065051325\_11252-model5 | -11.3433 |
|  | XP\_065051325\_11252-model5.1 | -11.317 |
|  | XP\_065051325\_11252-model5.2 | -11.317 |
| # cluster 6 - Size: 13 |  |  |
|  | XP\_065051325\_11252-model6 | -11.3074 |
|  | XP\_065051325\_11252-model6.1 | -11.3074 |
|  | XP\_065051325\_11252-model6.2 | -11.3064 |
|  | XP\_065051325\_11252-model6.3 | -11.3052 |
|  | XP\_065051325\_11252-model6.4 | -11.3052 |
|  | XP\_065051325\_11252-model6.5 | -11.3052 |
|  | XP\_065051325\_11252-model6.6 | -11.3052 |
|  | XP\_065051325\_11252-model6.7 | -11.3052 |
|  | XP\_065051325\_11252-model6.8 | -11.212 |
|  | XP\_065051325\_11252-model6.9 | -11.212 |
|  | XP\_065051325\_11252-model6.10 | -11.0741 |
|  | XP\_065051325\_11252-model6.11 | -11.0741 |
|  | XP\_065051325\_11252-model6.12 | -11.0741 |
| # cluster 7 - Size: 9 |  |  |
|  | XP\_065051325\_11252-model7 | -11.3019 |
|  | XP\_065051325\_11252-model7.1 | -11.3019 |
|  | XP\_065051325\_11252-model7.2 | -11.3019 |
|  | XP\_065051325\_11252-model7.3 | -11.3019 |
|  | XP\_065051325\_11252-model7.4 | -11.3019 |
|  | XP\_065051325\_11252-model7.5 | -11.3019 |
|  | XP\_065051325\_11252-model7.6 | -11.3019 |
|  | XP\_065051325\_11252-model7.7 | -11.265 |
|  | XP\_065051325\_11252-model7.8 | -11.2075 |
| # cluster 8 - Size: 4 |  |  |
|  | XP\_065051325\_11252-model8 | -11.2064 |
|  | XP\_065051325\_11252-model8.1 | -11.1317 |
|  | XP\_065051325\_11252-model8.2 | -11.1013 |
|  | XP\_065051325\_11252-model8.3 | -11.0006 |
| # cluster 9 - Size: 1 |  |  |
|  | XP\_065051325\_11252-model9 | -11.1681 |
| # cluster 10 - Size: 6 |  |  |
|  | XP\_065051325\_11252-model10 | -11.1659 |
|  | XP\_065051325\_11252-model10.1 | -11.1659 |
|  | XP\_065051325\_11252-model10.2 | -11.0901 |
|  | XP\_065051325\_11252-model10.3 | -11.0172 |
|  | XP\_065051325\_11252-model10.4 | -10.9462 |
|  | XP\_065051325\_11252-model10.5 | -10.7147 |
| # cluster 11 - Size: 3 |  |  |
|  | XP\_065051325\_11252-model11 | -11.1414 |
|  | XP\_065051325\_11252-model11.1 | -10.9494 |
|  | XP\_065051325\_11252-model11.2 | -10.9458 |
| # cluster 12 - Size: 1 |  |  |
|  | XP\_065051325\_11252-model12 | -11.1041 |
|  |  |  |
